# Effectiveness of provider-initiated versus client-initiated HIV testing by different health facility departments in Northern Tanzania

**DOI:** 10.1186/s12981-023-00541-z

**Published:** 2023-07-07

**Authors:** Ramadhani Abdul, Tobias F. Rinke de Wit, Giulia Martelli, Kathleen Costigan, Patrobas Katambi, Peter Mllacha, Anton Pozniak, Werner Maokola, Sayoki Mfinanga, Sabine Hermans

**Affiliations:** 1 Amsterdam UMC, Department University of Amsterdam, Department of Global Health, Amsterdam Institute for Global Health and Development, Amsterdam, the Netherlands; 2 Bugisi Health Centre, Shinyanga, Tanzania; 3 Infectious Diseases Unit, AUSL Romagna, Morgagni Pierantoni Hospital Forlí, Doctors with Africa CUAMM IT, Forlí, Italy; 4 Ngokolo Health Centre, Shinyanga, Tanzania; 5 Shinyanga Regional Referal Hospital, Shinyanga, Tanzania; 6 Chelsea and Westminster Hospital NHS Foundation Trust and LSHTM, London, UK; 7 National Aids Control Program(NACP), Dodoma, Tanzania; 8 National Institute for Medical Research(NIMR)-Muhimbili centre, Dar es Salaam, Tanzania; 9 Alliance for Africa Health Research, Nairobi, Kenya; 10 School of Public Health, Department of Epidemiology and Statistics, Muhimbili University of Health and Allied Science, Dar es Salaam, Tanzania

**Keywords:** Provider initiated testing and counselling(PITC), Client initiated Counselling and Testing(CITC), HIV testing, Tanzania

## Abstract

**Background:**

HIV prevalence in Tanzania is still high at 4.7% among adults. Regular HIV testing is consistently advocated in the country to increase the level of awareness of HIV status, thus contributing to national HIV prevention. We report findings from three years of implementation of an HIV Test and Treat project utilizing provider-initiated and client-initiated testing and counselling (PITC and CITC). This study compared the effectiveness of PITC versus CITC in HIV case detection by the different departments of health facilities.

**Method:**

This retrospective cross-sectional study used health facility-based HIV testing data collected from adults aged 18 years and above between June 2017 – July 2019 in the Shinyanga region, Tanzania. Chi-square and logistic regression analysis were used to assess determinants of yield (HIV positivity).

**Results:**

A total of 24,802 HIV tests were performed of which 15,814 (63.8%) were by PITC and 8,987 (36.2%) by CITC. Overall HIV positivity was 5.7%, higher among CITC at 6.6% than PITC at 5.2%. TB and IPD departments had the highest HIV positivity 11.8% and 7.8% respectively. Factors associated with a positive test were testing at a department in the facility compared to CITC, first-time test, and being or having been married compared to being single.

**Conclusion:**

Success in identifying HIV + patients was highest among people visiting the clinic for HIV testing (CITC) and first-time testers. With PITC, HIV + patient detection differed between departments, suggesting divergent risk profiles of respective clients and/or divergent HIV alertness of staff. This underscores the importance of increased targeting for PITC to identify HIV + patients.

## Introduction

Despite progress made, the prevalence of HIV in Tanzania remains high. The latest 2020 UNAIDS estimates the prevalence in adults aged 15–49 at 4.5(4.2–4.6)%, about 88(85–96)% of people living with HIV know their HIV status, and the proportion on ART is estimated at 86(83–93)% [1, 2]. In 2016–2018 a population-based survey estimated the first 90 of the 90-90-90 goals, awareness of HIV status among those estimated to be HIV-positive, at 60.6% [3]. Since then, Tanzania has adopted multiple approaches to increase HIV case detection, which includes Provider-initiated Testing and Counselling (PITC), whereby health providers recommend HIV testing services (HTS) to all clients seeking health services [4]. Despite the Tanzania National Guideline recommending providers to offer PITC [5], its general implementation is still low [6]. Although PITC alone is unlikely to yield the desired testing coverage on its own [7], its contribution is considered important [8, 9], and suboptimal use would be a missed opportunity to diagnose and link HIV patients into care [10, 11]. Continuing evaluation is essential to understand how PITC is implemented, the characteristics of people accessing the services, and the corresponding HIV testing yields across the facility.

This study aims at assessing the effectiveness of PITC in HIV case detection by the different departments of health facilities (tuberculosis, outpatient, and inpatient department) and comparing this with Client Initiated Testing and Counselling (CITC), in which the client reports at the facility to get tested for HIV. Specifically, the study aimed to (a) describe the testing and sociodemographic characteristics of people coming forward for testing in each of the facilities, overall and by testing strategy (PITC and CITC); (b) determine the testing yield by testing strategy (PITC vs. CITC), overall and stratified by the facility, (c) calculate PITC yield in different departments within the facility and (d) analyse factors associated with finding HIV positive patients.

## Method

### Study setting and design

This retrospective cross-sectional study used routine facility-based historical data on HIV testing from testing registers of three facilities: Shinyanga Regional Referal Hospital (SRRH) and Ngokolo Health Centre (Ngokolo HC) in the municipal district, and Bugisi Health Centre (Bugisi HC) in a rural district of Shinyanga region, between June 2017 and July 2019. Bugisi HC and Ngokolo HC are private, faith-based facilities. SRRH is a public regional referral facility.

This study was nested within the Shinyanga & Simiyu Test and Treat study, which assesses the feasibility of universal access to HIV Test and Treat by implementing a differentiated HIV care model in North-Western Tanzania [12]. SRRH was only added as a project facility in 2020 and therefore did not receive staff support during the study period. As part of the study, several testing approaches were implemented: community-based testing and facility-based testing [13]. For this paper, we only report on facility-based testing.

### Facility-based HIV testing approaches

Two testing approaches were investigated. First, CITC, whereby a client voluntarily attends the facility for HTS at a dedicated CITC department within the facility. As per national guidelines, repeat testing was not done if a client returned within three months. Secondly, PITC, an HIV test offered to all clients visiting different departments (i.e., outpatient department (OPD), inpatient department (IPD), prevention of mother to child transmission (PMTCT), and tuberculosis (TB)) within the facility. If agreed, the testing is done within the department; if no qualified staff is available or for any other reason, a client can be referred to another department for testing. Regardless of the actual testing place, client information is captured in the respective department register where the testing was initiated. The National HIV testing guideline utilizes a serial testing approach, using Point of Care Tests (POCT); the first test uses the SD-Bioline test and the second test uses UniGold-HIV rapid test if the first test is reactive.

### Participants

All clients recorded in the HIV testing registers aged one year or above in the three facilities from June 2017 to July 2019 were included in the study. This period was chosen as the start date of the Test and Treat Project up to the introduction of a new HTS by the Government in 2020. This study included only adults aged 18 years or above.

### Data sources, detail of measurements, and definitions

Data came from individual-level HIV testing registers from the participating facilities. HIV testing registers from all departments of the facility were retrieved, and data were extracted and entered into the National HTS data entry program by trained data clerks.

Demographic variables included were age at testing (in years), sex, and marital status of the clients. Other variables included pregnancy status for women, date of testing, the facility department where HIV testing was initiated, type of counselling given (client alone, client and parents, couple or group), type of test (new or repeat), testing type (PITC or CITC), and HIV test result (positive or negative). Testing data for the TB department were only available at SRRH as they were included in the overall OPD data for the other two facilities. Testing data for PMTCT were only available at Bugisi HC and were evaluated as part of PITC.

For this study, the residence of clients (urban or rural) was assigned as per the testing facility department: SRRH and Ngokolo HC as urban clients, while Bugisi HC as rural clients. This was done due to lack of information on clients’ address in the testing register. Repeat tests were defined as tests performed for clients who self-reported at least one previous HIV test before the current one regardless of their previous HIV test result. First-timers were the clients who had never tested before as self-reported. The self-reported question was used to determine repeat testing as persons could not be linked to any previous testing register entries because no personal identifiers are recorded in the registers.

### Data analysis

Data were analyzed using Stata 15 software. Frequencies and percentages described categorical variables and means (SD) or medians (IQR) for continuous variables. Chi-square tests were used to compare the testing yield between different characteristics of clients receiving services stratified by testing strategy. Univariable and multivariable logistic regression models were employed to analyze the risk factors associated with HIV positivity (confounders were defined *a priori*). To allow for clustering within facilities, we used robust standard errors. To assess if there may have been misclassification of CITC-originated testing as PITC testing at SRRH; a sensitivity analysis without the SRRH was performed. A p-value of 0.05 was considered to be significant for all analyses.

## Results

### Descriptive analysis of testing, overall and by testing strategy (PITC and CITC)

Overall, 24,802 tests were performed during the study period, 63.8% using PITC and 36.2% using CITC. Table 1 describes the characteristics of study participants by testing type. Overall, more females (53.2%) than males tested for HIV, and males tested more often through CITC than PITC (50.6% versus 44.7%, p < 0.001). There was a difference in counselling type between CITC and PITC: the proportion of clients who received couple counselling was twice as high among CITC (15.9%) than PITC (8.9%). The proportion of first testers was higher among PITC clients (22.4%) than CITC (18.6%), p < 0.001. Of all the PITC clients, the vast majority of tests were initiated in the OPD (84.4%), followed by IPD (12.4%) and the TB department (2.3%).

**Table 1:** Participants’ characteristics, overall and by testing type

Characteristics (n (%)	Overall n = 24,802(100%)	PITC n = 15,814 (63.8%)	CITC n = 8987 (36.2%)	P-Value*
**Gender**				
Female Male	13,181(53.2) 11,620(46.8)	8746(55.3) 7068(44.7)	4435(49.4) 4552 (50.6)	< 0.001
**Age in years (mean (SD)**	34.8(14.6)	36.5(15.7)	31.8(11.7)	< 0.001
**Age**				
18–25 years 25–50 years 50 years and above	7666(30.9) 13,741(55.4) 3394 (13.7)	4400(27.8) 8727(55.2) 2687(16.9)	3394(36.3) 5014(55.8) 707(7.9)	< 0.001
**Pregnancy status (n = 15,794)**				
Yes No	101(0.8) 12,113(99.2)	61(0.8) 7988(99.2)	40(1.0) 4125(99.0)	0.241
**Marital status**				
Single Married/cohabiting union Separated/divorced Widow	5803(23.4) 17,071(68.8) 1282(5.2) 645()2.6)	3163(20.0) 11,404(72.1) 750(4.7) 497(3.1)	2640(29.4) 5667(63.1) 532(5.9) 148(1.6)	< 0.001
**Facility Name**				
Bugisi HC Ngokolo HC SRRH	16,653(67.1) 2328(9.4) 5820(23.5)	10,354(65.5) 735(4.7) 4725(29.9)	6299(70.1) 1593(17.7) 1095(12.2)	< 0.001
**Residence**				
Rural Urban	16,653(67.1) 8148(32.9)	10,354(65.5) 5460(34.5)	6299(70.1) 2688(29.9)	< 0.001
**Prior Testing History**				
first test Repeat test	5206(20.9) 19,595(79.1)	3538(22.4) 12,276(77.6)	1668(18.6) 7319(81.4)	< 0.001
**Department within the facility**				
CITC IPD OPD TB** Other	8987(36.2) 1966(7.9) 13,340(53.8) 371(1.5) 137(0.6)	8987(100) - - - -	- 1966(12.4) 13,340(84.4) 371(2.4) 137(0.9)	N/A
**Counselling type**				
Client alone Client and parents Couple Group	21,397(86.2) 309(1.2) 2849(11.5) 246(0.9)	14,049(88.8) 230(1.4) 1419(8.9) 116(0.7)	7348(81.8) 79(0.9) 1430(15.9) 130(1.4)	< 0.001

* Comparing proportions between PITC and CITC **Only SRRH had information from the TB department separately (others within OPD). Legend: PITC- Provider Initiated Testing and Counseling and CITC-Client initiated testing and counselling, SRRH-Shinyanga Regional and Referal Hospital

**Fig. 1:**
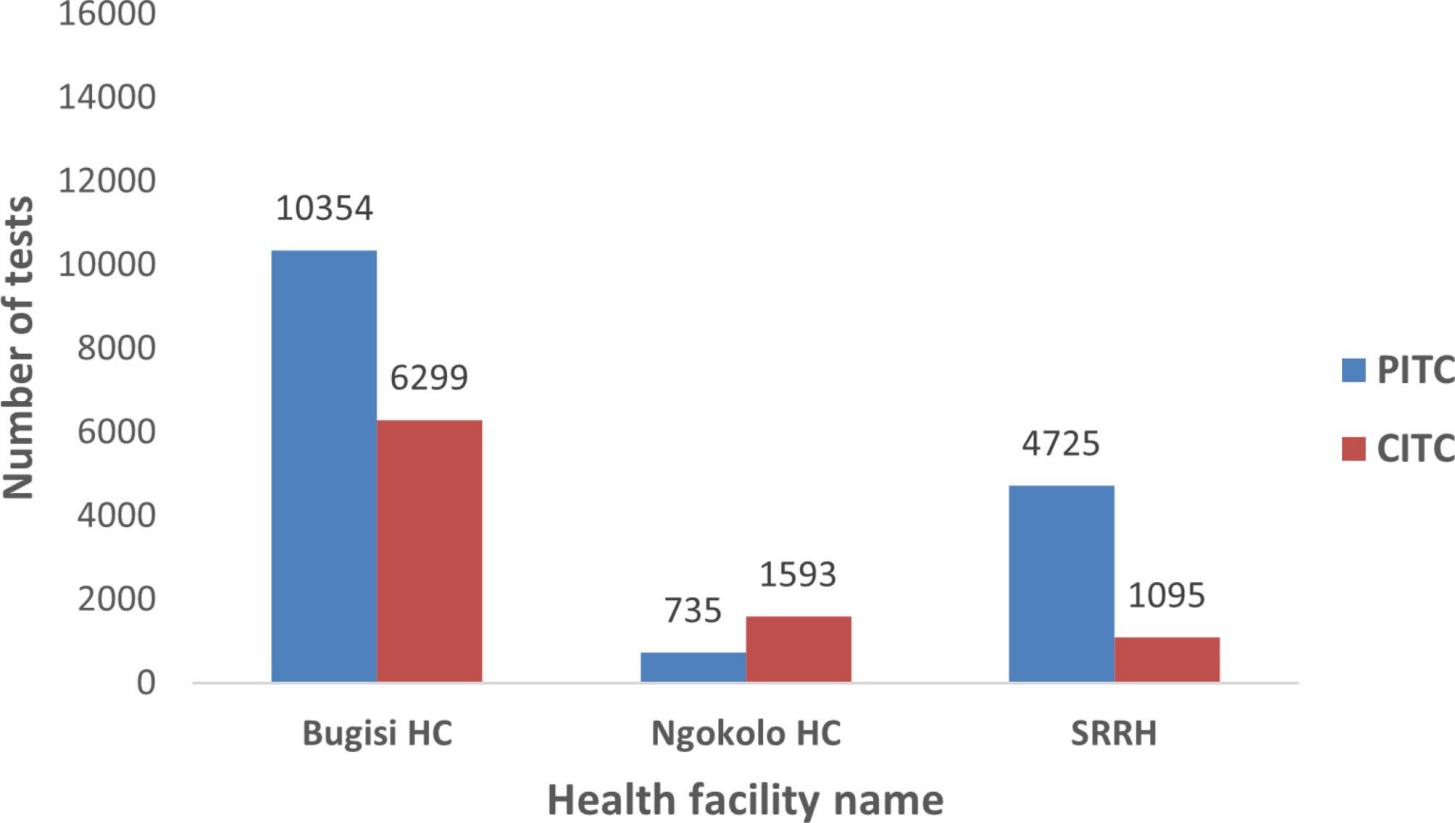
Testing numbers of CITC and PITC by facility Legend: PITC-Provider Initiated Testing and Counseling and CITC-Client initiated testing and counselling, SRRH-Shinyanga Regional and Referal Hospital

Figure 1 presents the HIV testing numbers by facility, comparing PITC and CITC. Most of the tests conducted at SRRH were done via PITC (81.2%), and CITC only accounted for (18.8%) of all HIV tests. A similar trend was observed in the Bugisi HC where the distribution of HIV testing was 37.8% by CITC and 62.2% by PITC. A reverse trend was observed at Ngokolo HC, whereby the majority of the tests were done through CITC (68.4%), and PITC only accounted for a third of all tests (31.6%). When further splitting testing numbers by the department in the clinic across all facilities, the majority of PITC tests originated from OPD (84.4%) followed by IPD (12.4%).

### Socio-demographic profile of PITC clients attending different testing departments in the facility

Comparing the socio-demographic profile of PITC clients who attended different departments in the facility. Males were more likely than females to receive HIV testing from the TB department (53% males) than from the IPD (34% males) or OPD (46% males). A slightly higher proportion of older adults (aged 50 years or more) tested at the TB department (24.5%), compared to 19.9%, 16.5%, and 7.9% of those tested at IPD, OPD, and CITC, respectively. The majority of clients who tested at IPD (71.7%) and OPD (73.0%) were married or cohabitating. Compared to other departments, the proportion of first-time testers was higher among those tested at IPD (35.6%) than at OPD (20.2%), TB (29.4%), or CITC (18.6%).

### HIV positivity yield by testing strategy (PITC vs. CITC), overall and stratified by facility

Table 2 presents HIV positivity yield by testing strategy. Of the 24,801 HIV tests performed, 1414 (5.7%) were found to be HIV positive. The positivity was higher among clients who tested through CITC (6.6%) than PITC (5.2%, p < 0.001). First-time testers had significantly higher HIV positivity than repeat testers (9.9% versus 4.6%). This difference was more pronounced among clients who tested through CITC (11.5% versus 5.5%, respectively) than PITC (9.1% versus 4.1%). Yield by PITC department was highest in the TB department (11.9%), followed by IPD (7.8%), OPD (4.6%), and lastly, other PITC departments (2.1%). When testing yields were further disaggregated by time, the HIV positivity decreases over time (overall, 6.6% in 2017, 5.5% in 2018 to 5.3% in 2019).

**Table 2:** HIV positivity yield (N and % of all tested) by testing strategy (PITC vs. CITC), overall

Characteristics (N (%)	Overall 1414(5.7)	PITC 820(5.2)	CITC 594(6.6)
**Gender**			
Female Male	787(5.9) 627(5.4)	449(5.1) 371(5.2)	338(7.6) 256(5.6)
**Age**			
18–25 years 25–50 years 50 years and above	231(3.0) 950(6.9) 233(6.9)	112(2.5) 550(6.3) 158(5.2)	119(3.6) 400(8.0) 75(10.6)
**Marital status**			
Single Married/cohabiting union Separated/Divorced Widowed	231(3.9) 843(4.9) 247(19.2) 93(14.2)	112(3.5) 512(4.5) 141(18.8) 55(11.1)	331(5.8) 106(19.9) 119(4.5) 38(25.7)
**Facility Name**			
Bugisi HC Ngokolo HC SRRH	851(5.1) 165(7.1) 398(6.8)	457(4.4) 43(5.9) 320(6.8)	394(6.2) 122(7.7) 78(7.1)
**Residence**			
Urban Rural	563(6.9) 851(5.1)	363(6.6) 457(4.4)	200(7.4) 394(6.2)
**Counselling type**			
Client alone Client and parents Couple Group	1166(5.5) 39(12.6) 197(6.9) 12(4.9)	690(4.9) 19(8.3) 106(7.5) 5(4.3)	476(6.5) 20(25.3) 91(6.4) 7(5.4)
**Department within the facility**			
CITC IPD OPD TB Other	594(6.6) 153(7.8) 620(4.6) 44(11.8) 3(2.1)	- 153(7.8) 620(4.6) 44(11.8) 3(2.1)	594(6.6) - - - -
**Prior testing history**			
Repeat test First test	901(4.6) 513(9.9)	499(4.1) 321(9.1)	402(5.5) 192(11.5)

**Legend: PITC- Provider Initiated Testing and Counseling and CITC-Client initiated testing and counselling, SRRH-Shinyanga Regional and Referal Hospital**

### Result of sensitivity analysis

The results from sensitivity analysis to assess if the misclassification of CITC clients who would have been classified as PITC at SRRH have shown no significant difference in the area under the curve for the models with and without SRRH at p = 0.63.

### Factors associated with HIV positivity

**Fig. 2:**
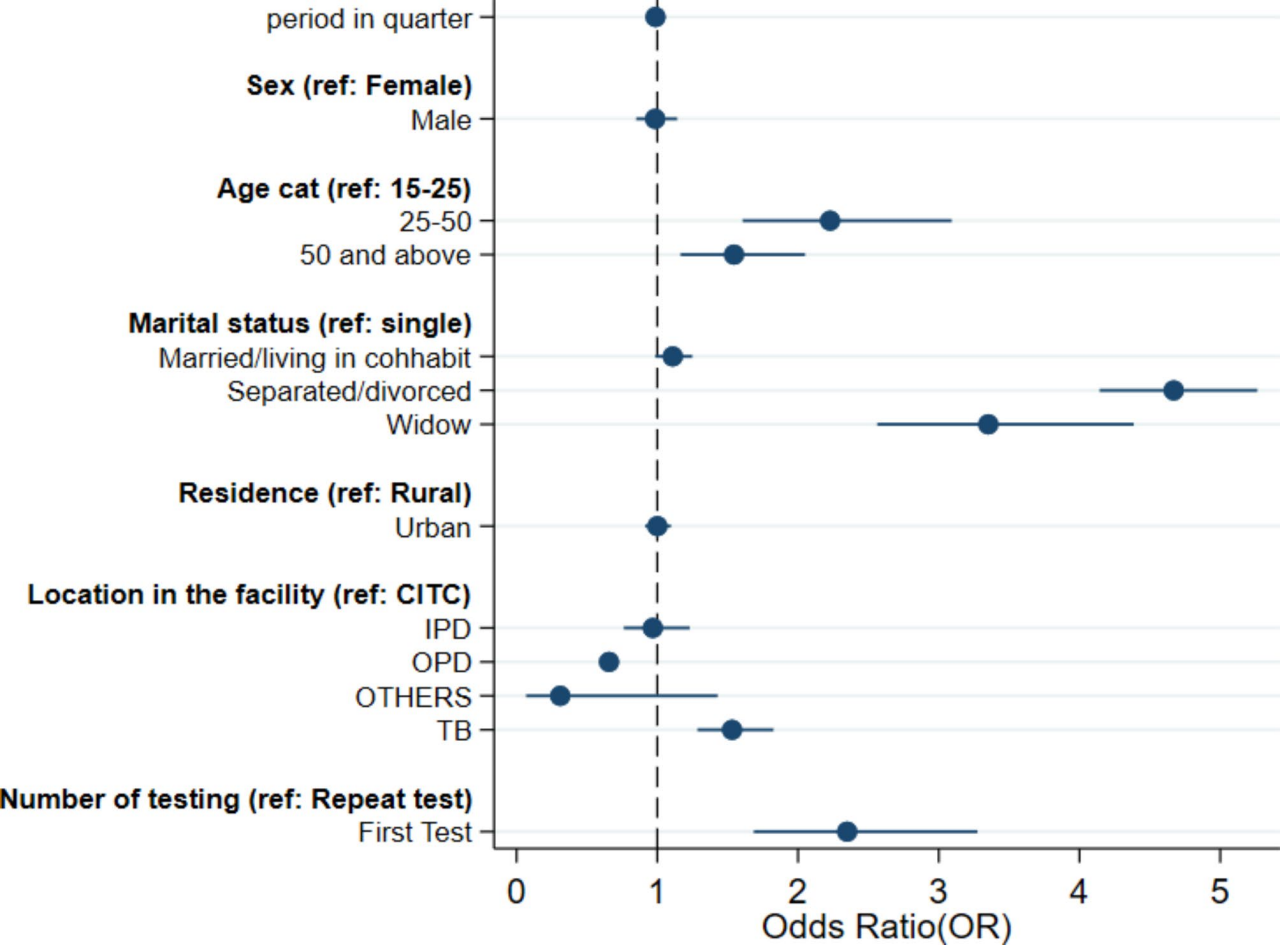
Adjusted Odds Ratio (OR) for being tested HIV positive Legend: PITC-Provider Initiated Testing and Counseling and CITC-Client initiated testing and counselling, IPD-Inpatient, OPD-Outpatient

Figure 2 presents the Odds Ratios (OR) of the factors associated with HIV positivity. These included age, marital status, department of testing initiation within the facility, and first-time testing. Being separated or divorced and being widowed was associated with a significantly increased likelihood of testing positive compared to being single (4.7-fold and 3.4-fold increase, respectively). The risk of a positive HIV test result was twice as among first-time testers compared to repeat testers.

The department where HIV testing was initiated within the facility was also associated with HIV positivity: when grouping all departments under PITC, the odds of HIV positivity were lower (OR = 0.63, 95% CI (0.43–0.90)) in PITC than in CITC. When comparing CITC with different departments of PITC separately, the clients whose testing was initiated at the TB clinic had a significantly higher risk of being positive (OR 1.53, 95%CI: 1.29–1.82). Clients in OPD were less likely to test positive, and clients in IPD had the same likelihood as CITC. There was no association with residence, the time period since the start of the study, or gender.

## Discussion

Overall HIV positivity among those tested through PITC and CITC in a large Test and Treat program in the Shinyanga region of Tanzania was 5.7%. HIV positivity was higher for CITC tests compared to PITC (6.6% vs. 5.2%) and was associated with older age, being separated/divorced, and testing for the first time.

Significant variation in the number and proportion of HIV tests by strategy and by health facility was observed. PITC accounted for 82%, 62%, and 32% of all tests done in SRRH, Bugisi HC, and Ngokolo HC, respectively. The higher proportion of PITC at SRRH is likely due to the unavailability of CITC services on some days of the week, which could be due to less project assistance compared to the other two facilities. Verbal communication with the facility in charge of Ngokolo HC revealed a limited number of trained staff and poor knowledge of PITC among clients as a possible reason for low PITC uptake in the facility. This demands further research to understand the actual reasons. Studies done in Tanzania assessing barriers to implementing PITC have listed a lack of staff training on PITC, limited testing equipment, a large number of patients, and a shortage of healthcare workers [14, 15].

The HIV prevalence we found among adults (5.7% in those aged 18 years or older) is comparable to the one reported from a population survey among adults 15 years or older (5.9%) done in this region [3]. Evidence from a systematic review of studies done in Sub-Saharan Africa [7] showed an overall higher prevalence found in facility-based studies than in the general population. Our finding of comparable prevalence estimates in the current study could suggest undertesting in the population visiting the facility. An alternative explanation could be that the majority of HIV-positives in the population have already been identified, and high-risk “pockets” remain who might not come forward for testing using routine approaches.

Studies have found consistent results in terms of the effectiveness of PITC in increasing testing rates [8, 9, 16]. However, its effectiveness in HIV case detection is less clear [9, 17, 18]. Unlike studies on PITC from a general health facility [17, 18] or OPD only [9], this study included various departments within health facilities, which were found to be attended by clients with distinct demographic profiles, which could differentially influence PITC HIV case finding rates.

Our study also compared HIV positivity in different departments of PITC in the facility, which revealed striking differences. Compared to CITC, the TB department of the PITC had the highest case detection, whereas it was lower in OPD, and there was no difference with the IPD. A recent study in Rwanda also showed no difference in HIV case detection between OPD and general facility attendees [9]. Our findings suggest a different HIV risk profile for people seeking services at different physical departments within a health facility. An alternative explanation could be different healthcare staff HIV alertness or guidelines per department. The high risk of HIV positivity among presumptive TB patients is well known [19–21]. The evidence provided in this study shows a need for a more focused approach that targets high-risk departments within facilities (TB clinics, in-patient departments).

Our finding of higher HIV positivity in CITC than PITC is contrary to other studies done in similar settings [8, 18, 22]. There are several explanations for this, the most likely being that PITC uptake was not as high as it could have been due to a lack of staff availability, training, or knowledge, as described above. Alternatively, lower PITC HIV prevalence could also be explained by the high number of repeat testers (79%); repeat testing has been associated with low(er) HIV prevalence [23]. Last, there could have been misclassification of CITC as PITC due to CITC staff shortages and/or underreporting of testing which could potentially affect the true PITC prevalence. Also, contrary to the population-based survey [3], this study did not find a significant difference in HIV prevalence between urban and rural populations. The use of facility location as a proxy for urban-rural classification could be the reason, since some of the clients from rural areas might have received HIV testing services from urban facilities and vice versa.

The main limitation of this study is that we could not estimate coverage and uptake of PITC in the facilities, as these data were not included in the testing registers and were not available to us at the time of the study. Comparing characteristics between those who accepted testing to those who refused would have been important to understand the magnitude of the PITC scale-up and the generalisability of our results (the extent of selection bias). PITC utilization information in the TB departments was only collected in SRRH while misclassified as OPD at other facilities, which likely overestimated HIV prevalence at OPD of these facilities and underestimated the magnitude of the risk factor of being tested in TB clinics. The results from SRRH need to be interpreted with caution as there was no project support to improve HTS during the study period. Although community sensitization campaigns may have led to increased visits to CITC, the lack of staff likely reduced the availability of services, and preferential reporting of HIV-positive cases, biasing toward a higher yield. There may have been misclassification of CITC-originated testing as PITC testing; a sensitivity analysis without the SRRH data did not change our findings, however, and SRRH as a government referral hospital would have incurred higher costs for CITC visits compared to the other two study facilities, which could also partly explain the lower CITC numbers. A limitation of the study design or data was that we were not able to analyze individuals, just tests. The authors used facility location as a proxy for clients’ residence; this could have led to misclassification and therefore lack of an association found. The data used for this analysis were from government testing registers, some important information such as why people sought CITC services would be of value but were not collected.

## Conclusion

Success in identifying HIV-positive patients was highest among CITC and first-time testers, and for PITC differed between departments at the facility, indicating divergent risk profiles of respective clients and/or divergent HIV alertness of staff. This underscores the importance for PITC of increased targeting to identify HIV-positive patients at high-risk departments within facilities (TB clinics, in-patient departments).

## Data Availability

These data were obtained from the Tanzania National Aids Control Program (NACP), after receiving permission from the Permanent Secretary of the Ministry of Health of Tanzania. Access to the data was limited to the conduct of relevant analysis and publication of results. A request to access the full data can be made to the NACP through the Ministry of Health by writing to the permanent secretary via email at ps@afya.go.tz.

## List of Abbreviations

CITC: Client Initiated Testing and Counselling
HC: Health Centre
HIV: Human Immunodeficiency Syndrome
HTS: HIV testing Services
IPD: Inpatient Department
IQR: Inter-Quartile Range
NIMR: National Institute for Medical Research
OPD: Outpatient Department
OR: Odds Ratio
PITC: Provider Initiated Testing and Counselling
PMTCT: Prevention of Mother to Children Transimission
POCT: Point of Care Test
SD: Standard Deviation
SRRH: Shinyanga Regional Referal Hospital
TB: Tuberculosis
UNAIDS: United Nations Programme on HIV/AIDS (UNAIDS)
